# Case of Phlegmonic Inflammation, with Reflections on Certain Effects of Heat and Cold on the Living System

**Published:** 1793

**Authors:** Thomas Beddoes


XIII. *Caſe of Phlegmonic Inflammation, with Reflections on certain Effects of Heat and Cold on the living Syſtem. By* Thomas Beddoes, *M. D.*

A PATIENT lately mentioned to me, among the particulars of her complaint, a circumſtance which ſeems, both on account of its ſingularity and of the illuſtration it affords of an important principle in animal nature, to be worth recording. The caſe was one of thoſe very common caſes where a ſmall irregularity in in diet, expoſure to cold, &c. produced pain and diſorder in the bowels, ſometimes ariſing to a ſevere fit of the colic. The patient having one day occaſion to waſh ſome butter, conceived that by removing her hands occaſionally out of the cold ſpring water into warm water, ſhe ſhould have a better chance of eſcaping the accuſtomed complaint in her bowels. She accordingly heated ſome water as hot as ſhe could well bear it, and, from time to time, transferred her arms out of the cold into the hot water, immerſing them pretty deep in the latter. It was on a Saturday in ſpring: the next morning ſhe was awakened by violent pain under each axilla, and was likewiſe ſenſible of a conſiderable ſwelling under each. The inflammation continued, and by Tueſday morning the tumors had increaſed, as ſhe ſays, to the ſize of a twopenny loaf each. They ſoon afterwards broke, and diſcharged a large quantity of pus. In about a fortnight both wounds were healed. Theſe circumſtances indicate a true phlegmonic inflammation, which, I ſuppoſe, may be ſafely aſcribed to the alternate action of heat and cold.

I know not whether it has been obſerved that the inflammations, particularly thoſe of the eyes, which are ſo frequent in thoſe hot climates where it is the cuſtom to ſleep during the ſummer in the open air, are to be referred to the ſucceſſion of heat to cold. Travellers, eſpecially thoſe into Egypt, have variouſly attempted to account for this phenomenon. Haſſelquiſt imputes it to certain miaſmata ariſing from the almoſt empty reſervoirs in which the water of the Nile is preſerved from inundation to inundation. This is, however, a mere hypotheſis, unconfirmed by any ſtrict analogy; nor is the ſuppoſed cauſe in any way brought home to the effect. As little, in my opinion, can the inflammation of the eyes be aſcribed to the influence of the nocturnal light of the heavens upon the eye, the eyelids being more or leſs cloſed during ſleep. The cauſe ſeems inadequate. It is common in this country to ſleep in chambers not leſs ſtrongly illuminated (if not more ſo) than in Egypt during the night, without any inconvenience to our ſight. Beſides, I think, if we could ſuppoſe the eye to be ſo dazzled by the light of the night as to be injured, the injury ought to fall upon the nerve and not upon the eyelids and external parts. The nitrous particles with which Alpinus imagines the atmoſphere of Egypt to be impregnated will not, I ſuppoſe, be conſidered as a cause more probable than any of the preceding: but the following passage may serve to give an idea of the nature of the complaint in question, and its frequency at Cairo. "Plurimasque (oculorum lippitudines) Cayri easdemque per omnia anni tempora homines invadere ob nitrosum pulverem, qui continuè oculos habitantium mordicat, et calefacit, observatur, longè maximéque in æstatis primâ parte, quo tempore calor ambientis summé calidi oculos inflammat, taliumque morborum numerum auget. *Sparsim* vero per urbem toto anno hæ oculorum inflammationes vagantur; atque *epidemicæ plurimæ* in primâ æstatis parte calidissimâ inæqualissimâque ob vehementissimum * meridionalium ventorum calorem, atque inflammatarum arenarum copiam, quæ ab iisdem ventis asportantur. Eo enim anni tempore è centum hominibus quinquaginta saltem lippientes observantur." (De Medicin. Ægypt p. 24.). The flying sand must be troublesome, and probably, in many cases, supports and increases the inflammation, and in some may give rise to it: but the following

* See Niebuhr's Thermometrical Tables in the first volume of his Travels.

fact, which seems to me to render the induction complete, shows that the true and general cause is the great inequality between the temperature of the night and day; to which cause signal effect is given by the practice of sleeping *sub dio*.

Mr. Clarkson (in his Essay on the impolicy of the African slave trade) informs us, (p. 71) that "when the slaves are brought on board, the seamen, to make room for them, are turned out of their apartments between decks, and sleep, for the most part, either on the deck or in the tops of the vessel during the whole of the middle passage; or from the time of their leaving the coast of Africa, (where the days are excessively hot, and the dews excessively cold and heavy, *ibid.* p. 68) to that of their arrival at the West-India islands." "From this bad lodging—he proceeds—and this continual exposure to colds and damps, and suddenly afterwards to a burning sun, fevers originate, which carry many of them off! Nor is this the only effect which this continual vicissitude from heat to extreme dampness and cold has upon the surviving crew: inflammatory fevers necessarily attack them. This fever attacks the whole frame: the eye feels the inflammation most. This inflammation terminates either in dispersion or suppuration: in the first instance inſtance the eyes are ſaved; in the latter, they are loſt."

The inflammation of the eye is not the only diſeaſe produced in Egypt by the ſucceſſion of hot days to cool nights any more than on board our ſlave ſhips; in both ſituations cauſes and effects run parallel, as the reader will find upon recurring to Alpinus and the later travellers. The well-known danger of expoſure to dews in hot climates, and indeed in all climates, in certain caſes, ſeems to depend on the ſame principle. It is alſo probable that the heat of the preceding day enables the dews of the night to prepare the ſyſtem for the ſtimulating effects of the heat of the ſucceeding day; ſo that of two perſons who ſhould expoſe themſelves without precaution to the cold of night and the heat of the following day, he who ſhould have been moſt exhauſted the day before by the heat, would, if other circumſtances could be rendered alike equal, be moſt injured by the next alternation.

Several circumſtances, ſuch as the redneſs and ſwelling of parts expoſed to cold, together with the frequent occurrence of inflammatory diſorders not long after expoſure to cold, were calculated to miſlead obſervers into a belief that theſe theſe diſorders were the direct effect of cold. Yet the great difference in the ſtate of a part during inflammation, and under the influence of cold, might have induced them to ſuſpect that ſo ſlight an analogy might be illuſive: and after taking into the account other well-aſcertained facts, they ought to have concluded that the theory was falſe. Linnæus, in a paper in the Amœnitates Academicæ, expreſſes his aſtoniſhment at the impunity with which the heated Laplander rubs himſelf with ſnow, or even rolls in the ſnow, and drinks the cold ſnow-water. We every day ſee horſes in a ſtate of the moſt profuſe perſpiration freely waſhed with cold water, and always without injury. I have ſeveral times within theſe two years cauſed horſes, accuſtomed to be ſtabled, to be turned out for a ſingle night in winter: and no cough, catarrh, or other diſorder, has ever been the conſequence. It appears therefore to me that, within certain limits, and thoſe not very narrow, the tranſition from a higher to a lower temperature is attended with no danger to animals in a ſtate of tolerable health; and a perſon, I conceive, might ſuddenly paſs from an higher to a lower temperature without inconvenience, even where the difference is ſo great as to to be capable of producing confiderable inflammation, if the change fhould be made with equal celerity in a contrary direction. On this, though an interefting fubject for obfervations on man, and experiments on animals, we want precife facts; and I ftate the principle in order to induce obfervers to compare with it the facts that fall in their way.

Befides the fucceffion of heat to cold, and *vice verfa*, there is a third cafe well worthy of confideration; and this where part of the body is expofed to one of thefe powers, and the remaining part to the other; as, for inftance, where a ftream of comparatively cold air flows upon part of the body of a perfon fitting in a warm room, and perhaps alfo drinking ftimulating liquors. In making chemical experiments it often happens that a cold (catarrh) is taken, if the hands be much immerfed in cold water, when the elaboratory is much heated; by adding warm water to raife the temperature of that in the trough this danger is eafily avoided. In thefe cafes the effect feems to be the fame as that of the fucceffion of heat to cold. In perfons whofe bowels are extremely liable to be affected, it fometimes happens, as I have myfelf known it to happen, that the removal of a foot into a cold part of the bed, after the body has become warm in bed, ſhall bring on acute pain in the bowels; and yet no pain is produced in getting into bed, though the temperature be the ſame, and perhaps lower, than that of the part into which the foot is removed; and probably total immerſion in cold water would not produce any pain in the bowels. The laws of ſuch phenomena, however deſerving of inveſtigation, have as yet ſcarcely been an object of attention with pathologiſts. It is probable that the phenomena, in any given caſe, are regulated by two circumſtances: firſt, by the exceſs of the heat (or the ſtrength of the ſtimulus, whatever it be) to which the greater part of the body is expoſed, above that to which the ſmaller is expoſed. 2. The ſecond circumſtance is the difference between the extent of the heated and cooled ſurfaces. When the latter is not extremely minute, and yet confined within moderate limits, the inflammatory effect ſeems to be conſiderable.

Should the circumſtances be reverſed, and a ſtream of air ſo warm as to convey heat to the body, inſtead of carrying it away, play upon a ſmall part of its ſurface, the reſt being expoſed to a moderate, or a low temperature, it is probable bable the refult would be the fame as when moderate cold fucceeds to warmth ; *i. e.* no bad effect would follow.

But I have added thefe latter fpeculations with a view to fhow how little has yet been afcertained concerning the operation of powers to whofe influence we are every moment expofed ; and undoubtedly we cannot expect the defideratum to be fupplied, till obfervers are rendered fully fenfible of its exiftence.

